# Does hyperglycemia affect arginine metabolites in critically ill patients? A prospective cohort and in vitro study

**DOI:** 10.1186/s13098-023-01035-8

**Published:** 2023-04-01

**Authors:** Tien F. Lee, Sara Tommasi, Andrew Bersten, Leonie K. Heilbronn, Salvatore Sotgia, Angelo Zinellu, Ciriaco Carru, Arduino A. Mangoni, Morton G. Burt

**Affiliations:** 1 College of Medicine and Public Health, Flinders University, Flinders Drive, Bedford Park, SA 5042 Australia; 2 Southern Adelaide Diabetes and Endocrine Services, Flinders Medical Centre, Flinders Drive, Bedford Park, SA 5042 Australia; 3 Department of Clinical Pharmacology, Flinders Medical Centre, Southern Adelaide Local Health Network, Bedford Park, SA 5042 Australia; 4 Department of Critical Care Medicine, Flinders University, Bedford Park, South Australia Australia; 5 Faculty of Health and Medical Sciences, The University of Adelaide, Adelaide, Australia; 6 Department of Biomedical Sciences, School of Medicine, University of Sassari, Sassari, Italy

**Keywords:** Stress hyperglycemia ratio, Asymmetric dimethyl-l-arginine, l-homoarginine, Endothelial function, Critical illness, Dimethylarginine-dimethylaminohydrolase 1

## Abstract

**Background:**

Changes in the arginine metabolites asymmetric dimethyl-L-arginine (ADMA) and L-homoarginine and acute blood glucose concentrations have been shown to cause endothelial dysfunction and be independently associated with mortality in Intensive Care Unit (ICU) patients. The aim of this study was to investigate whether hyperglycemia potentially modulates these arginine metabolite concentrations to provide a mechanism that may link hyperglycemia and mortality in this patient group.

**Methods:**

A clinical and in vitro study were undertaken. Glucose, glycosylated hemoglobin-A1c (HbA1c) and the stress hyperglycemia ratio (SHR) (to quantify absolute, chronic and relative hyperglycemia respectively) were measured in 1155 acutely unwell adult patients admitted to a mixed medical-surgical ICU. SHR was calculated by dividing the admission glucose by the estimated average glucose over the last 3 months, which was derived from HbA1c. ADMA and l-homoarginine were measured in a plasma sample collected at admission to ICU by liquid chromatography tandem mass spectrometry. The activity of dimethylarginine-dimethylaminohydrolase 1 (DDAH1), the main enzyme regulating ADMA concentrations, was assessed at varying glucose concentrations in vitro by quantifying the conversion of ADMA to citrulline in HEK293 cells that overexpress DDAH1.

**Results:**

In the clinical study, plasma ADMA was not significantly associated with any measure of hyperglycemia. L-homoarginine was positively associated with glucose (β = 0.067, p = 0.018) and SHR (β = 0.107, p < 0.001) after correction for glomerular filtration rate. However, as l-homoarginine is a negative predictor of mortality, the direction of these associations are the opposite of those expected if hyperglycemia was affecting mortality via changes in l-homoarginine. In vitro DDAH1 activity was not significantly influenced by glucose concentrations (p = 0.506).

**Conclusion:**

In critically ill patients the association between relative hyperglycemia and mortality is not mediated by changes in ADMA or L-homoarginine.

*Trial registration* ANZCTR Trial ID ACTRN12615001164583.

## Background

Hyperglycemia in critically ill patients is associated with increased mortality and morbidity [1–5]. Furthermore, the relative increase in blood glucose during critical illness is more strongly associated with mortality than absolute hyperglycemia, and this relationship is independent of other variables that predict prognosis in Intensive Care Unit (ICU) patients [6]. Similar findings have been reported in patients with an acute myocardial infarction [7]. However, these observations do not confirm that relative hyperglycemia contributes to mortality directly or provide a mechanism linking hyperglycemia and mortality.

Several plausible pathogenic mechanisms have been postulated to link hyperglycemia with adverse outcomes [8–12]. Both direct and indirect effects have been implicated. Indirect effects include electrolyte imbalances, hypoperfusion, volume depletion or acid–base imbalance. Direct effects previously studied include oxidative stress, inflammation, induction of apoptosis, activation of the coagulation cascade, and impaired endothelium-dependent vasodilation due to endothelial dysfunction [13–16]. Endothelial dysfunction is present in septic patients in ICU [17, 18], and is an independent predictor of mortality in critically ill patients [19].

Endothelial dysfunction is often assessed by quantifying limb blood flow following localized ischemia [13, 20]. However, this is impractical in large cohorts of critically ill patients. An alternative approach to assess endothelial function is measurement of l-arginine and related metabolites in plasma. Asymmetric dimethyl-l-arginine (ADMA), symmetric dimethyl-l-arginine (SDMA), monomethyl-l-arginine (MMA), and l-homoarginine (HA) directly or indirectly regulate the activity of the enzyme endothelial nitric oxide synthase (eNOS). This enzyme converts l-arginine to citrulline and the potent endogenous vasodilator nitric oxide (NO), which also has anti-inflammatory, anti-thrombotic and anti-atherosclerotic effects [21].

Decreased NO production has been associated with increased cardiovascular morbidity and mortality in patients with cardiovascular disease and hypertension [22, 23]. We have previously demonstrated that ADMA is positively and l-homoarginine negatively associated with increased mortality in patients with an acute critical illness [24]. These changes in arginine metabolites have also been reported by others and are associated with a reduction in endothelial NO production [25, 26]. Acute hyperglycemia is a well-recognized cause of endothelial dysfunction [27]. Moreover, previous studies in a rodent model have reported that the activity of the main enzyme that regulates ADMA concentration in plasma, dimethylarginine-dimethylaminohydrolase 1 (DDAH1), is down-regulated by hyperglycemia [28]. This would increase ADMA concentration, consequently reducing NO production, and potentially provide a mechanistic link between acute hyperglycemia, endothelial dysfunction and mortality [8].

We hypothesized that acute hyperglycemia in critically ill patients reduces DDAH1 activity, thereby increasing plasma ADMA concentration and reducing endothelial-dependent vasodilatation. To investigate this we have undertaken: (1) a clinical study investigating whether measurements of glycemia are associated with arginine metabolites in critically ill patients; and (2) an in vitro study assessing the effect of glucose concentration on DDAH1 activity.

## Methods

### Subjects

For the clinical study, we studied the patient cohort recruited to assess the relationship between relative hyperglycemia and mortality in critically ill patients, as described previous in detail [6]. In brief, we prospectively included consecutive medical and surgical ICU admissions at Flinders Medical Centre, Adelaide, Australia, between 27 January 2016 and 30 March 2017 that met inclusion and exclusion criteria. Subjects were excluded if they had previously been admitted to ICU within the study period or were admitted for routine post-operative monitoring following a surgical procedure, aged < 18 years, pregnant or had a recent blood transfusion, were admitted primarily for treatment of hyper- or hypoglycemia or had missing data preventing calculation of risk of death score. In the remaining subjects, we stored the plasma sample collected closest to admission to ICU at − 70 °C for subsequent measurement of arginine and metabolites. If no plasma sample was collected within 24 h of admission to ICU, the subject was excluded from this analysis.

### Arginine metabolites

Arginine and its chemically related metabolites and analogues MMA, L-homoarginine, ADMA and SDMA were measured according to the method developed by Sotgia et al. [29]. The method has previously been described in detail [24]. Aliquots of plasma were spiked with 1 µL of internal standard solution containing L-homoarginine-d4 dihydrochloride (d4-hArg), NG,NG-dimethyl-L-arginine-d6 dihydrochloride (d6-ADMA), and NG,NG’-dimethyl-l-arginine-d6 (d6-SDMA). After the addition of 400 µL of ultrapure water (Milli-Q grade), heat-treatment and centrifugation, a 200-µL volume of clear supernatant was combined with 20 µL of potassium phosphate monobasic buffer (100 mmol/L, pH 7.0) and 40 µL of diethylpyrocarbonate (33 mmol/L) for analysis using liquid chromatography tandem mass spectrometry (LC/MS–MS). Mass detection was accomplished in positive ion mode by MRM of the precursor-product ion transitions *m/z* 247.14 → 142, 261.28 → 70, 261.28 → 84, 275.33 → 46, and 275.33 → 70 for arginine, MMA, homoarginine, ADMA, and SDMA, respectively, as well as *m/z* 265.28 → 88, 281.3 → 52, and 281.3 → 70 for d4-homoarginine, d6-ADMA, and d6-SDMA, respectively.

### Absolute and relative hyperglycemia

To quantify absolute glycemia, venous plasma glucose at admission to ICU was measured on a Roche P modular analyzer (Hitachi High-Technologies Corporation, Tokyo, Japan) using the hexokinase/glucose-6-phosphate dehydrogenase assay (between-run CV 1.7% at glucose 4.9 mmol/L and 1.4% at glucose 15.7 mmol/L). Relative hyperglycemia was quantified using the Stress Hyperglycemia Ratio (SHR). SHR was calculated by dividing the admission glucose by the estimated average glucose over the prior 3 months, which was derived from HbA1c [30]. HbA1c was measured by high-performance liquid chromatography (PDQ; Primus Diagnostics, Kansas City, MO) using boronate affinity chromatography (between-run coefficient of variation [CV] 2.2% at HbA1c 6.1% [43 mmol/mol] and 1.9% at HbA1c 11.1% [98 mmol/ mol]).

### DDAH1 activity

In the in vitro study, cell lysate prepared from HEK293T cells overexpressing DDAH1 was used to investigate the direct effect of glucose on DDAH1 activity [31]. An established and robust isotope-dilution ultra-performance liquid chromatography couple to mass spectrometry (UPLC-MS)-based DDAH1 activity assay was used to measure the conversion of ADMA to citrulline [32].

All analyses were performed in triplicate. HEK 293 T cell lysate, which overexpressed DDAH1 at a concentration of 0.4 mg/mL, was pre-incubated in a 0.1 mol/L phosphate buffer containing glucose at concentrations of 0, 5, 7.5, 10, 15, and 22.5 mmol/L for one hour before adding 45 µmol/L of ADMA (equivalent to Km) to initiate the reaction. After 30 min, the reaction was terminated by adding acidified propanol (0.1% v/v formic acid in 2-propanol), followed by the addition of the assay internal standard, l-citrulline-d6, and centrifugation to remove the precipitated proteins. The supernatant fraction was then concentrated by evaporation and reconstitution, and the concentration of citrulline formed during the incubation was measured by UPLC-MS. l-citrulline was separated on a Waters ACQUITY UPLC BEH HILIC column (1.7 µm, 2.1 mm × 100 mm) at a flow rate of 0.3 mL/min using a gradient mobile phase containing 0.1% v/v formic acid and acetonitrile in water. The concentrations of l-citrulline were measured using an Acquity UPLC system (Waters, Sydney, Australia) coupled with a quadrupole time-of-flight (qToF) Premier high-resolution mass spectrometer (Waters, Sydney, Australia) in positive MS mode. The selected ion chromatograms were extracted from the total ion chromatogram at m/z 176.10 → 159.10 and 182.13 → 165.12, which correspond to the fragments of L-citrulline and L-citrulline-d6, respectively.

### Statistical analysis

Subject characteristics are presented as mean ± standard deviation if the distribution was normal or median (interquartile range) if they were not normally distributed. Comparisons between variables in patients who survived and died were assessed using unpaired *t*-tests or the Mann–Whitney *U*-test as appropriate. Simple linear regression analyses were used to compare the relationship between measures of glycemic control and arginine metabolites. As all variables were not normally distributed they were log-transformed to attain a normal distribution before the statistical analysis. As some arginine metabolites are renally cleared, these odds ratios were then assessed for independence from glomerular filtration rate (GFR) in a multiple linear regression analysis to ensure that differences in renal function were not underlying results. Finally, the effect of glucose concentration on DDAH1 activity was assessed using one way analysis of variance.

Statistical analysis was undertaken using SPSS version 25 for Windows (IBM, New York, USA); A two-tailed P-value of < 0.05 was considered statistically significant.

## Results

### Subject characteristics

There were 1262 critically ill patients recruited for the study investigating the relationship between relative hyperglycemia and mortality in critically ill patients [6]. In this cohort, 107 patients did not have an adequate plasma sample for arginine metabolomics studies and excluded from further analysis. Therefore, 1,155 patients were included in this analysis. (Table 1). These patients had a wide range of diagnoses with 207 patients admitted under Cardiology or Cardiothoracic Surgery, 112 under Respiratory Medicine, 113 under Neurology or Neurosurgery, 30 under Renal Medicine, 32 under Hematology or Oncology, 22 under Psychiatry, 77 under Hepatology or Hepatobiliary Surgery, 146 under other surgical specialities and 416 under General Medicine or other medical specialities. In the cohort, 163 patients (14.1%) died while in hospital or within 3 months of a hospital transfer. Patients that died were older, admitted for non-surgical reasons, had a higher glucose, SHR, ADMA, MMA and SDMA and a lower EGFR, arginine and L-homoargine (Table 1). Other clinical details are previously described in detail [24].

**Table 1:** Demographics, glycemia and arginine metabolite concentrations of critically ill patients who survived and died

Characteristic	Survivors	Deceased	p-value
Number	992	163	
Age (years)	61 ± 18	70 ± 15	< 0.001
Male sex (%)	56	58	0.671
Surgical admission (%)	34	16	< 0.001
Glucose (mmol/L)	7.4 (6.0–9.4)	8.9 (6.5–12.4)	< 0.001
HbA1c (%) (mmol/mol)	5.7 (5.4–6.5) 38.8 (35.5–47.5)	5.8 (5.3–6.3) 39.9 (34.4–45.6)	0.273
SHR	1.10 (0.89–1.35)	1.34 (1.02–1.84)	< 0.001
EGFR (ml/min/1.73 m^2^)	67 (38–96)	45 (24–72)	< 0.001
ADMA (µmol/L)	0.54 (0.45–0.66)	0.60 (0.49–0.79)	< 0.001
Arginine (µmol/L)	61.7 (42.9–83.4)	53.8 (32.8–81.7)	0.005
Homoarginine (µmol/L))	0.88 (0.55–1.36)	0.65 (0.35–1.16)	< 0.001
MMA (nmol/L)	65.0 (50.8–82.6)	76.7 (57.1–106.2)	< 0.001
SDMA (µmol/L)	0.69 (0.51–1.12)	0.85 (0.60–1.45)	< 0.001

Values are mean ± standard deviation or median (interquartile range) if not normally distributed. Comparison between groups used unpaired *t*-tests for normally distributed variables and a Mann-Whitney *U*-test if the distribution was not normal

*HbA1c* glycosylated hemoglobin A1c; *SHR* stress hyperglycemia ratio; *EGFR* estimated glomerular filtration rate; *ADMA* asymmetric dimethyl-l-arginine; *MMA* monomethyl-l-arginine; *SDMA* symmetric dimethyl-l-arginine

### Arginine metabolites in critical illness

L-homoarginine was weakly but significantly positively associated with HbA1c and SHR in univariate analyses and with glucose and SHR after correction for GFR (Table 2). SDMA was significantly associated with glucose, HbA1c and SHR in univariate analyses. After correcting for GFR, the correlations with glucose and SHR remained statistically significant (Table 2). ADMA, arginine, and MMA were not significantly associated with any glucose parameter, either unadjusted or after correction for GFR (Table 2). GFR was associated with all arginine metabolites, an effect that was independent of all glucose parameters (data not shown).

**Table 2:** Simple and multiple linear regression analyses between measures of glycaemic control and arginine metabolites

	Glucose		HbA1c		SHR		Glucose*		HbA1c*		SHR*	
	R	P value	r	P value	r	P value	β	P value	β	P value	Β	P value
ADMA (nmol/L)	0.012	0.671	0.002	0.940	0.016	0.590	0.004	0.879	− 0.046	0.109	0.038	0.190
Homoarginine (nmol/L)	0.057	0.054	0.086	0.004	0.123	< 0.001	0.067	0.018	− 0.048	0.097	0.107	< 0.001
Arginine (µmol/L)	0.004	0.897	0.009	0.755	0.001	0.994	0.005	0.860	0.025	0.405	− 0.009	0.762
MMA (nmol/L)	0.028	0.335	0.025	0.391	0.15	0.613	0.021	0.468	− 0.017	0.559	0.036	0.206
SDMA (nmol/L)	0.132	< 0.001	0.068	0.021	0.189	< 0.001	− 0.153	< 0.001	− 0.035	0.105	− 0.139	< 0.001

*HbA1c* glycosylated hemoglobin A1c; *SHR* stress hyperglycemia ratio; *ADMA* asymmetric dimethyl-l-arginine; *MMA* monomethyl-l-arginine; *SDMA* symmetric dimethyl-l-arginine

^*^Corrected for log GFR. Log GFR is independently associated with arginine metabolite concentrations in all analyses

### DDAH1 activity

**Fig. 1:**
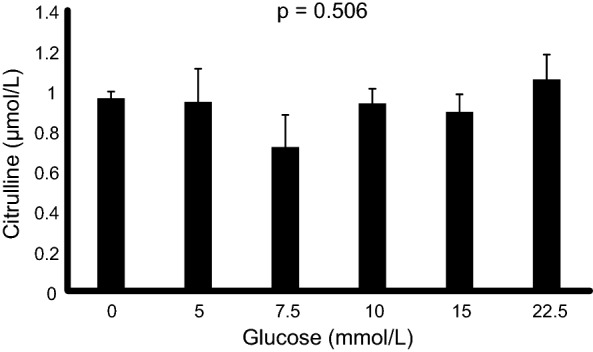
Effect of glucose on the conversion of ADMA to citrulline catalysed by DDAH1. Legend: Each data point represents the mean of three singlicate experiments (n = 3). Error bars represent the standard deviation. Statistical analysis was performed using analysis of variance

Citrulline production at different glucose concentrations in vitro is shown in Fig. 1. There was no significant effect of glucose concentration on citrulline production (p = 0.506).

## Discussion

We assessed the relationship between arginine metabolites and 3 different measurements of hyperglycemia (reflecting absolute, relative and chronic hyperglycemia) in a large cohort of critically ill patients. We found that there was no association between glucose parameters and plasma ADMA concentration. Concordant with these findings, differing glucose concentrations in vitro did not affect DDAH1 activity. There was a weak positive correlation between l-homoarginine and glucose parameters. However, as l-homoarginine is negatively associated with mortality [24], the direction of these associations is the opposite of that expected if hyperglycemia was affecting mortality via changes in l-homoarginine. Taken together, the findings strongly imply that hyperglycemia does not increase mortality by altering arginine metabolites.

In the clinical study there was not a significant association between ADMA and any of the 3 measures of hyperglycemia. As we had reported that relative hyperglycemia [6] and ADMA [24] were independently associated with mortality, we hypothesized that a glucose-mediated elevation in ADMA could be a mechanistic link between these two findings. Previous studies using non-invasive measures to assess endothelial function have reported that increasing ADMA [33] and blood glucose acutely both cause endothelial dysfunction [9, 27]. However, in this large cohort of critically ill patients there was no correlation between relative hyperglycemia and ADMA.

Our in vitro study was consistent with a lack of effect of acute hyperglycemia on ADMA concentration. Differences in glucose concentration across the range encountered in clinical practice did not affect DDAH1 activity, the main enzyme metabolizing ADMA and responsible for its plasma concentration. It was previously hypothesized that hyperglycemia causes endothelial dysfunction by down-regulating DDAH1 [8] and this theory was supported by data from a rodent model of chronic hyperglycemia [28]. It is likely that differences in the biological model and duration of hyperglycemia contribute to the differences between our study and that of Lin et al. [28]. Our combined clinical and in vivo data suggest that changes in plasma ADMA do not mediate the relationship between acute hyperglycemia and mortality in humans.

There was a weak but significant positive association between l-homoarginine and both glucose and SHR after correction for GFR. However, plasma l-homoarginine concentration was lower in patients who died than in survivors of critical illness and there was a negative association between l-homoarginine and mortality [6]. This is consistent with reports of a negative association between l-homoarginine and mortality in other patient groups [25, 34, 35]. Therefore, if the relationship between relative hyperglycemia and mortality was being mediated by changes in l-homoarginine, the association between l-homoarginine and SHR should be negative. As the association is weak and in the opposite direction, this suggests the relationship between acute hyperglycemia and mortality is not via modulation of l-homoarginine.

After correction for renal function, there was a weak but significant association between SDMA and glucose and SHR. It was previously postulated that SDMA may competitively inhibit arginine uptake at the cationic amino acid transporters hence reducing available arginine for NO synthesis [36]. A previous study in critical illness showed an association between mortality and SDMA but postulated that the association merely reflected the differences in renal clearance of SDMA [37]. SDMA was not an independent determinant of mortality in this cohort [6]. There was also not a significant association between arginine and MMA and any of the 3 glucose parameters. Taken with our previous results [24], it is unlikely that the relationship between relative hyperglycemia and mortality is mediated by SDMA, MMA or arginine.

Acute hyperglycemia has been demonstrated to cause changes in vascular tone and endothelial dysfunction that are associated with increased mortality in critically ill patients [8, 13, 38]. However, our results suggest that endothelial dysfunction in the critically ill is not mediated by changes in arginine metabolites. Given the negative findings in this study, it is possible that relative hyperglycemia: (1) is primarily linked to mortality by a different mechanism not related to endothelial dysfunction, and/or, (2) causes endothelial dysfunction through an arginine-independent pathway. We support further studies to elucidate mechanisms linking relative hyperglycemia and mortality.

This study has several notable strengths. It comprised a large prospective population of consecutive critically ill patients. The in vivo and in vitro studies results are internally consistent as both studies suggest that hyperglycemia does not affect arginine metabolites. However, there are several limitations of this study. The clinical study was observational, with inherent limitations, such as the inability to infer causality. Measurement of arginine metabolites was not the primary endpoint of the study. Another limitation is that only one mechanism by which arginine metabolites are modulated was assessed in vitro. However, as ADMA was the strongest independent predictor of mortality in critically ill patients, DDAH1 activity was considered the most important variable to study.

## Conclusion

In summary, relative hyperglycemia is not associated with plasma ADMA concentration in ICU patients and hyperglycemia does not alter the activity of ADMAs main regulator DDAH1. Relative hyperglycemia is weakly associated with L-homoarginine, but the direction of the association between L-homoarginine and SHR is the opposite to that expected if acute hyperglycemia was increasing mortality by altering L-homoarginine concentration. Hence, we conclude that in critically ill patients, the association between relative hyperglycemia and mortality is not mediated by changes in arginine metabolites.

## Data Availability

The datasets generated and/or analysed during the current study are not publicly available due to restrictions of the ethics approval but are available from the corresponding author on reasonable request.
